# Hybridization and recurrent evolution of left–right reversal in the land snail genus *Schileykula* (Orculidae, Pulmonata)

**DOI:** 10.1111/jzs.12353

**Published:** 2019-12-10

**Authors:** Josef Harl, Elisabeth Haring, Barna Páll‐Gergely

**Affiliations:** ^1^ Institute of Pathology University of Veterinary Medicine Vienna Austria; ^2^ Central Research Laboratories Museum of Natural History Vienna Austria; ^3^ Department of Integrative Zoology University of Vienna Vienna Austria; ^4^ Plant Protection Institute Centre for Agricultural Research Hungarian Academy of Sciences Budapest Hungary

**Keywords:** *12S*, *16S*, *COI*, histone genes, molecular phylogeny, taxonomy

## Abstract

The land snail genus *Schileykula* Gittenberger, 1983 is distributed in arid limestone areas from western Turkey to north‐western Iran. It comprises eight species, which display high variation in shell size and morphology. The cylindrical shells are 5–12 mm in height and the last shell whorls bear several inner lamellae and plicae. Two taxa differ in their chirality having sinistral shells, while all the others are dextrals such as the vast majority of orculids. The aim of this study was to establish a molecular genetic phylogeny of *Schileykula* and to test whether it conforms to the current morphology‐based classification. Furthermore, we were interested in the phylogenetic position of the two sinistral forms in order to assess whether one or two reversals happened in the evolution of the genus. Nine out of ten species, including all four subspecies of *Schileykula trapezensis* and three of six subspecies of *Schileykula scyphus*, were investigated. A section of the mitochondrial *cytochrome c oxidase subunit I* gene was analyzed in 54 specimens of *Schileykula* and from a subsample, partial sequences of the mitochondrial genes for the *12S rRNA* and the *16S rRNA*, and a section of the nuclear *H4/H3* histone gene cluster were obtained. The phylogenetic trees based on the mitochondrial sequences feature high support values for most nodes, and the species appear well differentiated from each other. The two chiral forms evolved independently and are not sister lineages. However, some groupings disagree with the present morphology‐based classification and taxonomical conclusions are drawn. *Schileykula trapezensis* is polyphyletic in the molecular genetic trees; therefore, three of its subspecies are elevated to species level: *Schileykula acampsis* Hausdorf, 1996 comb. nov., *Schileykula neuberti* Hausdorf, 1996 comb. nov., and *Schileykula contraria* Neubert, 1993 comb. nov. Furthermore, *Schileykula sigma* is grouped within *S. scyphus* in the mitochondrial and nuclear trees and consequently treated as a subspecies of the latter (*Schileykula scyphus sigma* Hausdorf, 1996 comb. nov.). *Schileykula nordsiecki*, whose shell morphology is indistinguishable from that of the neighboring *Schileykula scyphus lycaonica*, but who differs in its genital anatomy, was confirmed to represent a distinct lineage. The phylogenies produced by the mitochondrial and nuclear data sets are to some extent conflicting. The patterns differ concerning the grouping of some specimens, suggesting at least two independent hybridization events involving *S. contraria*, *S. scyphus* and *S. trapezensis*. The results exemplify the importance of integrating both mitochondrial and nuclear sequence data in order to complement morphology‐based taxonomy, and they provide further evidence for hybridization across distantly related lineages in land snails.

## Graphical abstract

The land snail genus *Schileykula* comprises eight species, distributed mainly in arid limestone areas in Turkey. We performed phylogenetic analyses based on three mitochondrial genes (*cytochrome c oxidase subunit 1*, *12S*, and *16S*) and a section of the nuclear *H4/H3* histone gene cluster. Some groupings disagree with the present morphology‐based classification, and taxonomical conclusions are drawn. *Schileykula trapezensis* is polyphyletic; therefore, three of its subspecies are elevated to species level. The two chiral forms evolved independently and are not sister lineages. The mitochondrial and nuclear data sets produced conflicting phylogenies, indicating at least two independent hybridization events.

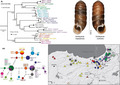

## 1 INTRODUCTION

The land snail genus *Schileykula* Gittenberger, 1983 is distributed in dry limestone areas of Asia Minor, from western Turkey to north‐western Iran (Hausdorf, 1996). Adults of *Schileykula* have cylindrical shells, which range from 5 to 12 mm in height. Shell shapes are less variable than the structure of inner lamellae and plicae in the adult shell. The morphology of the latter has thus been primarily used for taxonomy. Currently, ten species and 18 subspecies (including the nominate forms) are recognized in *Schileykula* as follows: *Schileykula aculeata* Gittenberger & Menkhorst, 1993, *Schileykula attilae* Páll‐Gergely, 2010, *Schileykula batumensis* (Retowski, 1889), *Schileykula inversa* Schütt, 1993, *Schileykula maculata* Páll‐Gergely & Asami, 2013, *Schileykula nordsiecki* Hausdorf, 1996, *Schileykula robusta* (Nägele, 1906), *Schileykula scyphus cilicica* Hausdorf, 1996, *Schileykula scyphus crassa* (Pilsbry, 1922), *Schileykula scyphus enteroplax* (Pilsbry, 1922), *Schileykula scyphus erecta* Hausdorf, 1996, *Schileykula scyphus lycaonica* Hausdorf, 1996, *Schileykula scyphus scyphus* (L. Pfeiffer, 1848), *Schileykula sigma* Hausdorf, 1996, *Schileykula trapezensis acampsis* Hausdorf, 1996, *Schileykula trapezensis contraria* Neubert, 1993, *Schileykula trapezensis neuberti* Hausdorf, 1996, and *Schileykula trapezensis trapezensis* (Stojaspal, 1981). The genital anatomy of almost all *Schileykula* species has been described and allows for distinguishing *Schileykula* from *Orculella* Steenberg, 1925 (Gittenberger, 1978; Hausdorf, 1996; Neubert, 1993; Páll‐Gergely, 2010, 2011; Páll‐Gergely & Asami, 2013; Páll‐Gergely & DeMattia, 2016). Although the generic position of *S. robusta* has not been confirmed based on its genital anatomy (no living specimens have been found so far), similarities in shell traits suggest placing it within *Schileykula* (see Hausdorf, 1996).

Current records show that most *Schileykula* taxa are distributed in a patchy, parapatric pattern, and different species usually do not co‐occur at the same localities but live in close vicinity. For example, *S. maculata* has only been recorded from a single site within the range of *Schileykula trapezensis* (Páll‐Gergely & Asami, 2013). *Schileykula nordsiecki*, which was found at a single locality between two disjunct populations of *S. s. lycaonica*, differs from the latter in its genital anatomy, while it is indistinguishable based on shell morphology (Hausdorf, 1996). *Schileykula attilae* and *S. batumensis* are known to inhabit opposite sides of the same castle hill (Páll‐Gergely, 2010). *Schileykula batumensis* and *S. t. acampsis* are the only *Schileykula* taxa known to occur in sympatry over a broad range, and the presence of populations with intermediate shell forms in the overlapping area was interpreted as an indication for hybridization between the two species (Neubert, 1993). Apart from the latter two taxa, potential hybrid populations were not reported for any other *Schileykula* taxa so far. However, the comparison of mt and nc sequence data in more recent studies showed that hybridization is not rare in land snails. There is evidence for hybridization between the orculid species *Orcula gularis* and *Orcula pseudodolium* (Harl, Páll‐Gergely, et al., 2014), and the mixing of genetically and morphologically extremely distinct populations of *Orcula dolium* (Harl, Duda, Kruckenhauser, Sattmann, & Haring, 2014). Evidence for introgressive hybridization between morphologically divergent land snails was found in the family Bradybaenidae in the genus *Mandarina* (e.g., Chiba, 2005) and between the genera *Ainohelix* and *Ezohelix* (Morii, Yokoyama, Kawata, Davison, & Chiba, 2015). RADseq data on the genus *Pyramidula* also provided evidence for minor ancestral gene flow between *Pyramidula pusilla* (Gittenberger & Bank, 1986) and *Pyramidula saxatilis* (Hartmann, 1842) (Razkin et al., 2016). Hybrid populations were investigated also in the genus *Albinaria* in Crete with SNP genotyping (Lammers et al., 2013). The presence of a hybrid population of the clausiliid species *Micropontica caucasica* (Schmidt, 1868) and *Micropontica circassica* (Boettger, 1888) in the Lagonaki plateau in the Caucasus, analyzed with AFLP markers, was interpreted as a case of hybrid speciation (Koch, Neiber, Walther, & Hausdorf, 2016).

A peculiarity of *Schileykula* is the presence of a sinistral (shell coiled counterclockwise) species and a sinistral subspecies, whereas all others are dextral (shell coiled clockwise). The sinistral subspecies (*S. t. contraria*) is known to occur between populations of the dextral *S. t. trapezensis*. *Schileykula inversa*, which is a sinistral species, lives close to populations of the dextral *S. s. erecta*, more than 200 km west of *S. t. contraria*. In gastropods reproducing by internal fertilization, the mating of snails with different chirality can cause genital mismatch (Asami, Cowie, & Ohbayashi, 1998; Gittenberger, 1988; Lipton & Murray, 1979). This mismatch is supposed to result in frequency‐dependent selection against the chirality type with lower frequency (Johnson, 1982). Yet, there are examples of chirally dimorphic populations of snail species (subgenus *Amphidromus*) that exhibit no difficulty of interchiral mating (Nakadera et al., 2010; Schilthuizen et al., 2007; Sutcharit, Asami, & Panha, 2007; Sutcharit & Panha, 2006). The role of chirality reversal in speciation, that is, whether different chiral types cause sexual isolation (“single gene speciation”), has been a matter of debate (Hoso et al., 2010; Richards et al., 2017; Ueshima & Asami, 2003; Yamamichi & Sasaki, 2013). Van Batenburg and Gittenberger (1996) investigated factors influencing the ease of fixation of a change in coiling direction. They found that the population size, number of invaders, and dominance of the mutant chirality gene are of higher importance than maternal effects and mobility. They also emphasize that the shift in coiling direction usually does not prevent mating in high‐spired snails, but that it often leads to reproductive isolation in snails with globular shells (Batenburg & Gittenberger, 1996). While dextral species in temperate regions are by far more common (often exceeding 99%), in Turkey, sinistral land snail taxa reach 5.5% (excluding the Clausiliidae which are mostly sinistral; Gittenberger, Hamann, & Asami, 2012). Five out of the 45 orculid taxa are sinistral: *Orculella heterostropha commagenensis* (Neubert, 1988), *Orculella heterostropha heterostropha* (O. Boettger, 1905) *Orculella menkhorsti sinistrorsa* Hausdorf, 1996, *S. inversa*, and *S. t. contraria*. So far, phylogenetic relationships of these taxa have not been investigated with molecular genetic methods. *Schileykula t. contraria* has been reported only from three close sites around which the dextral *S. t. trapezensis* occurs, raising the question of whether they are actually separate species.

In the present study, we tested the morphology‐based systematics of *Schileykula* by molecular phylogenetic analyses using DNA sequences of three mitochondrial (mt) genes and one nuclear (nc) sequence region. We included almost all extant species (except for *S. robusta*) and most subspecies of *Schileykula*. We also included several specimens of *Sphyradium doliolum* (Bruguière, 1792) from the monotypic genus *Sphyradium* Charpentier, 1837, the sister group of *Schileykula* (Harl et al., 2017). *Sphyradium doliolum* has a wide distribution from the Pyrenees in the west to northern Iran in the east, partially overlapping with that of *Schileykula* (Hausdorf, 1996).

Besides the establishment of a DNA‐based phylogeny of *Schileykula*, we addressed the following specific questions: (a) Is the polytypic *S. trapezensis*, with four morphologically distinct subspecies, monophyletic? (b) Is *S. nordsiecki* a part of the *S. scyphus* group or is it a distinct species as the anatomy suggests? (b) Did hybridization occur between nearby occurring *Schileykula* taxa in north‐eastern Turkey, the most speciose region within the range of the genus? (d) Do the two sinistral taxa represent independent lineages? That is, did a single reversal occur in the common ancestor of the two species or were there two independent reversals?

## 2 MATERIALS AND METHODS

### 2.1 Taxon sampling

We performed DNA analyses on 56 specimens of 14 *Schileykula* taxa. Only *S. robusta* and three subspecies of *S. scyphus*, *S. s. cilicica* Hausdorf, 1996, *S. s. crassa* (Pilsbry, 1922), and *S. s. erecta*, were not included. For out‐group comparison, we included 31 individuals of *Sp. doliolum* from a variety of places in its wide distribution including Austria, Hungary, Greece, Croatia, Romania, Slovenia, and Turkey. Before they were broken for tissue preparation, shells of all specimens were pictured with a WILD MAKROSKOP M420 and a NIKON DS Camera Control Unit DS‐L2 in frontal, lateral, apical, and umbilical view. Remaining parts of specimens are stored in the tissue collection of the Central Research Laboratories of the Natural History Museum Vienna (NHMW). Taxon names, individual identification IDs (IndIDs), and localities with GPS coordinates are listed in Table 1.

**Table 1:** Data on species, individuals, and localities

Species	Identification IDs	Locality [site no.]	WGS84 (N)	WGS84 (E)	m. asl
*Schileykula aculeata*	6256, **6257**	TR, Karadeniz, Bölgesi Artvin, Ardanuç, Ardanuç castle [**21**]	41°08.4′	42°00.5′	530
*Schileykula attilae*	**6254**	TR, Karadeniz, Bölgesi Artvin, Şavşat, Şavşat, Kalesi [**23**]	41°15.6′	42°19.6′	950
*Schileykula batumensis*	6619, **6620**	TR, Artvin, Yusufeli, Boarder to Erzurum, 1km S of Kınalıçam [**20**]	40°42.5′	41°40.7′	740
*Schileykula batumensis*	7116, 7117, **7118**	TR, Erzurum, Uzundere, Uzundere, 1km S of Uzundere [**19**]	40°31.0′	41°31.7′	1,120
*Schileykula maculata*	**7089**, **7090**	TR, Gümüşhane, Gümüşhane, Kale, Kale fortress [**7**]	40°23.2′	39°42.1′	1,581
*Schileykula nordsiecki*	**6594**, **6595**, 6596, 6597	TR, Konya, Bozkir, Bozkir, Bozkir [**2**]	37°11.4′	32°16.7′	1,370
*Schileykula scyphus enteroplax*	6630, **6631**	TR, Gümüşhane, Gümüşhane, Gümüşhane, 1km NW of Mescitli [**6**]	40°31.1′	39°24.9′	1,080
*Schileykula scyphus enteroplax*	**7088**	TR, Gümüşhane, Gümüşhane, Torul, 6km S of Torul [**5**]	40°31.8′	39°20.5′	980
*Schileykula scyphus inversa*	**6635**, 6636, **6637**	TR, Amasya, Amasya, Amasya, Amasya [**4**]	40°39.6′	35°50.9′	430
*Schileykula scyphus lycaonica*	**6599**, **6600**, 6601	TR, Kastamonu, Bozkir, Bozkir, 2,5km NE of Kizilçakir [**3**]	37°12.9′	32°35.5′	1,360
*Schileykula scyphus scyphus*	5490, **5491**	TR, Bursa, Bursa, Iznik, Besevler [**1**]	40°26.8′	30°02.8′	210
*Schileykula sigma*	**7108**, 7109, 7110	TR, Bayburt, Bayburt, Aşkale, 16km SW of Aşkale [**16**]	39°50.3′	40°34.1′	1,836
*Schileykula sigma*	**6258**, **6259**	TR, Erzinan, Tercan, Tercan, Tercan tunnel [**15**]	39°50.4′	40°34.0′	1,800
*Schileykula trapezensis acampsis*	**6616**, 6617, **6618**	TR, Erzurum, İspir, İspir, 3km SE of Çamlıkaya [**17**]	40°37.5′	41°11.6′	930
*Schileykula trapezensis acampsis*	7114, **7115**	TR, Erzurum, Uzundere, Engüzekappı kalesi, 2km S Uzundere [**18**]	40°30.6′	41°31.6′	1,200
*Schileykula trapezensis contraria*	**6608**, 6609, **6610**	TR, Bayburt, Bayburt, boarder to Erzurum, 12km S of Maden [**11**]	40°05.3′	40°25.4′	1,800
*Schileykula trapezensis contraria*	**7096**, **7097**	TR, Bayburt, Bayburt, Bayburt, NW of Çalıdere [**12**]	40°06.7′	40°25.4′	1,760
*Schileykula trapezensis contraria*	7098, **7099**	TR, Bayburt, Bayburt, Bayburt, 3km NW of Çalıdere [**13**]	40°07.2′	40°25.5′	1,750
*Schileykula trapezensis neuberti*	**6626**, 6627, **6628**	TR, Artvin, Ardanuç, Ardanuç, 1km S of Ardanuç [**22**]	41°07.7′	42°03.2′	530
*Schileykula trapezensis trapezensis*	**6613**, **6614**, 6615	TR, Bayburt, Bayburt, boarder to Erzurum, 6km S of Maden [**10**]	40°07.8′	40°25.1′	1,780
*Schileykula trapezensis trapezensis*	7091, **7092**, 7093	TR, Bayburt, Bayburt, Balkaynak, 2 km W of Balkaynak [**8**]	40°21.7′	39°53.1′	1,750
*Schileykula trapezensis trapezensis*	**7094**, 7095	TR, Bayburt, Bayburt, Balkaynak, 5 km N of Bayburt [**9**]	40°18.1′	40°13.8′	1,525
*Schileykula trapezensis trapezensis*	**7102**, 7103, 7104	TR, Bayburt, Bayburt, Maden, Kopdağ centre junction [**14**]	40°03.2′	40°27.0′	2,000
*Sphyradium doliolum*	833, **2803**, 2804	AT, Niederösterreich, Baden, Breitenstein, Adlitzgraben	47°39.4′	15°50.2′	650
*Sphyradium doliolum*	5443	HR, Primorsko, goranska županija, Cres, Cres, Lubenice	44°53.3′	14°20.6′	360
*Sphyradium doliolum*	5451	SI, Bovec, Bovec, Koritnica valley, Trdnjava Kluče	46°21.6′	13°35.6′	534
*Sphyradium doliolum*	2835, 2836, 2837	HU, Baranya, Komló, Manfa, Mecseki, erdö	46°08.8′	18°14.7′	322
*Sphyradium doliolum*	2839, 2840	HU, Baranya, Komló, Komló city, Sikonda reservoir	46°10.8′	18°13.1′	182
*Sphyradium doliolum*	2841, 2842, 2843	HU, Baranya, Komló, Mánfa city, Kolyuk	46°09.0′	18°12.6′	227
*Sphyradium doliolum*	**2845**, 2846, 2847	HU, Veszprém, Tapolcai kistérség, Balaton lake, Hertelendy monument	46°47.9′	17°29.9′	334
*Sphyradium doliolum*	**5460**, 5461	SI, Bovec, Bovec, Koritnica valley, Trdnjava Kluče	46°21.6′	13°35.6′	534
*Sphyradium doliolum*	5459	SI, Bovec, Bovec, Soča valley, Kobarid	46°14.8′	13°34.8′	260
*Sphyradium doliolum*	5462, 5463	AT, Kärnten, Klagenfurt Land, Loibltal, Tscheppa gorge	46°29.1′	14°15.8′	695
*Sphyradium doliolum*	**6577**, 6578, 6579	RO, Tulcea, Tulcea, Nifon, 6 km NW of Nifon	45°12.4′	28°19.9′	160
*Sphyradium doliolum*	**6581**, **6582**	RO, Tulcea, Tulcea, Cocoş Monastary, forest next to monastary	45°12.8′	28°24.4′	145
*Sphyradium doliolum*	**6588**	TR, Bursa, İnegöl, boarder to Bilecik, 1,5km E of Mezit	39°55.7′	29°43.9′	580
*Sphyradium doliolum*	**6593**	TR, Isparta, Uluborlu, Uluborlu, Uluburlo	38°04.8′	30°29.6′	950
*Sphyradium doliolum*	**7111**, 7112, 7113	GE, Samtskhe, Javakheti, Ozero Kakhisi Tba, 4.5 km S of Bakuriani	41°43.2′	43°29.7′	1,873

Note

**Figure 1:**
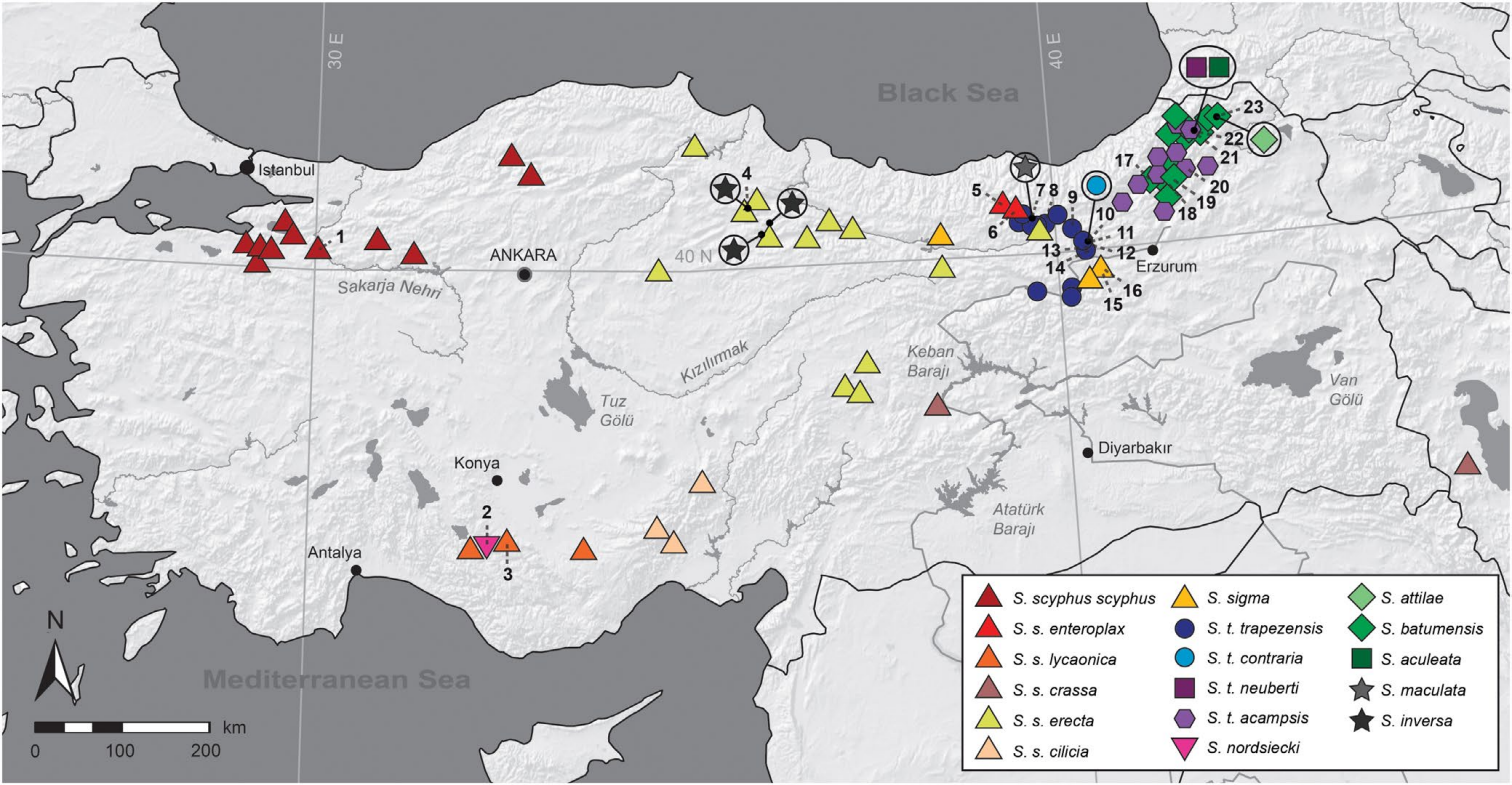
Distribution of *Schileykula* in Turkey and Iran. Numbers indicate sampling sites of the present study (see also Table 1)

**Figure 2:**
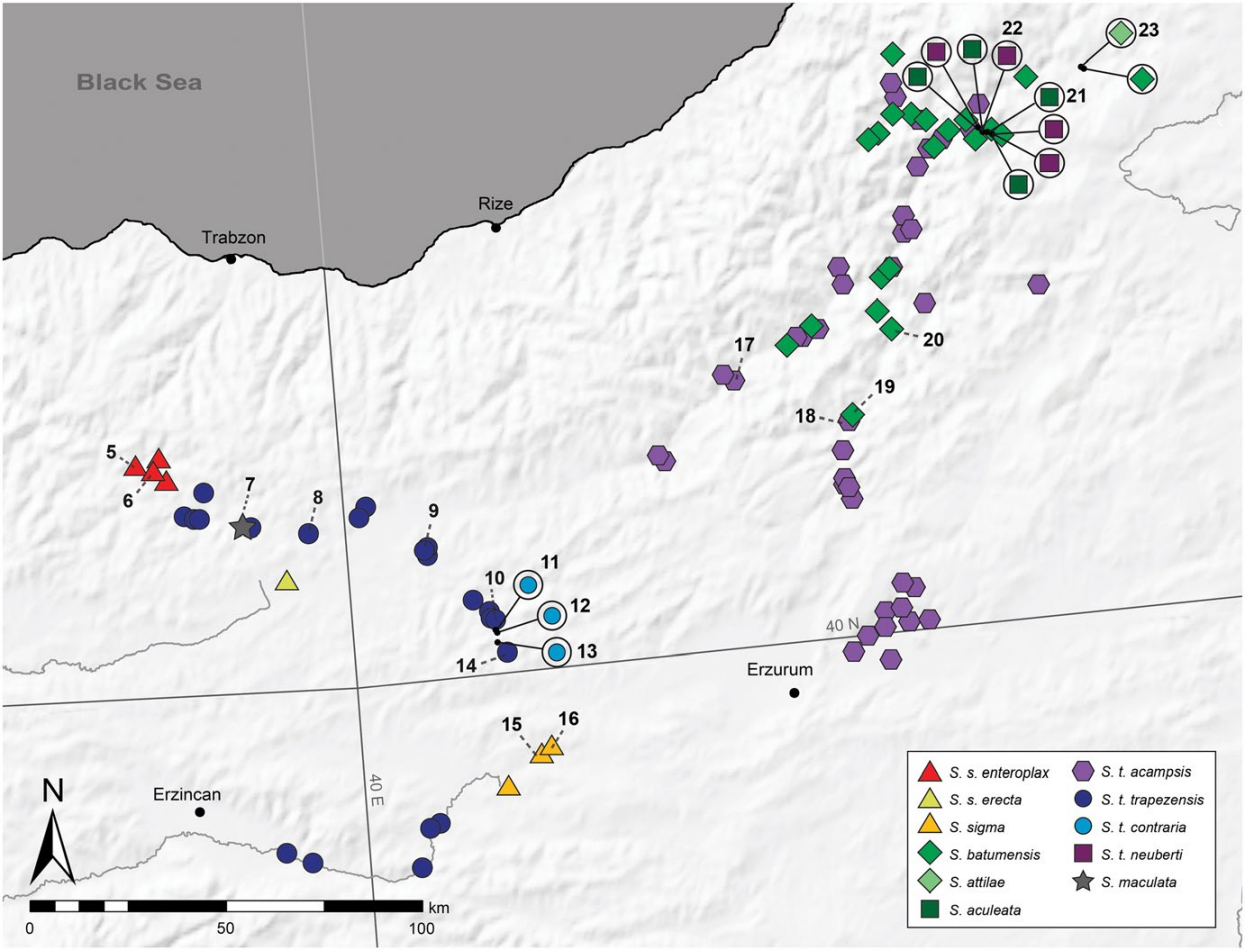
Distribution of *Schileykula* in eastern Turkey. Numbers indicate sampling sites of the present study (see also Table 1)

Specimens of which the whole data set. (*COI*, *12S*, *16S*, H4/H3) was obtained are indicated in bold individual numbers. Bold numbers in square brackets correspond to the numbers in Figures 1 and 2.

### 2.2 Distribution maps

Distribution maps of all *Schileykula* taxa were prepared using ArcMap Desktop 10.0 and manually edited in Adobe Photoshop CC v.2015.01 (Adobe Systems). Distribution data originate from Hausdorf (1996), Neubert (1993), and the present authors. Figure 1 shows a rough overview on the known localities of *Schileykula* taxa as well as the sampling sites of specimens investigated in the present study. A detailed view of distribution ranges in north‐eastern Turkey is provided in Figure 2.

### 2.3 DNA extraction and markers

Molecular phylogenetic analyses were performed with two to five individual specimens per species and locality (87 individuals in total). DNA was extracted from small pieces of foot tissue (ca. 1–2 mm in diameter) using the DNeasy Blood and Tissue Kit (QIAGEN) following the standard protocol for DNA extraction from tissue. DNA is stored at −80°C in the DNA collection of the Central Research Laboratories of the NHMW. A section of the mt *cytochrome c oxidase subunit 1* (*COI*) was amplified from 86 specimens. For a selection of 35 *Schileykula* and nine *Sphyradium* specimens, we additionally obtained partial sequences of the mt genes for the *12S ribosomal RNA* (*12S*) and the *16S ribosomal RNA* (*16S*), and a fragment of the nc H4/H3 gene cluster including major parts of the *histone H4* (*H4*) and *histone H3* (*H3*) as well as the complete non‐coding intergenic spacer region (IGS), which is positioned between *H4* and *H3*. Information on the primers (names, sequences, references, amplicon sizes) and annealing temperatures is listed in Table 2.

**Table 2:** Primers and annealing temperatures used in the present study

Region	Primer (5′–3′)	Origin	PCR product length (sites)	Annealing T (°C)
COI	LCO1490: GGTCAACAAATCATAAAGATATTGG	Folmer, Black, Heah, Lutz, and Vrijenhoek (1994)	706	50 (Taq)
	HCO2198: TAAACTTCAGGGTGACCAAAAAATCA	Folmer et al. (1994)		
12S	12SGastFw2: AGTGACGGGCGATTTGT	Cadahía et al. (2014)	706–746	50–54 (Taq)
	12SGastRv4: TAAGCTGTTGGGCTCATAAC	Cadahía et al. (2014)		
16S	16SLOrc1_fwd: TTACCTTTTGCATAATGGTTAAACTA	Harl, Duda, et al. (2014)	882–912	50–54 (Taq)
	16SLOrc_rev: CGGTCTGAACTCAGATCATG	Harl, Duda, et al. (2014)		
H4/IGS/H3	OrcH4_left1: GTGCGTCCCTGGCGCTTCA	Harl, Duda, et al. (2014)	1,125–1,186	55–57 (Taq)
	OrcH3_right1: TGGGCATGATGGTGACACGCT	Harl, Duda, et al. (2014)		71 (Phusion)

IGS, intergenic spacer.

### 2.4 PCR and cloning

PCRs for the mt markers (*COI*, *12S*, *16S*) were performed with the Roche Taq Polymerase (Roche) in 25 µl volumes with 0.2 mM of each dNTP, 1 mM of each primer, 3 mM MgCl_2_, 5 µl 10× PCR buffer, and Taq DNA Polymerase (1 U). PCRs started with 3 min at 94°C, followed by 35 cycles with 30 s at 94°C, 30 s at the respective annealing temperatures (Table 2), 1 min at 72°C, and a final extension for 7 min at 72°C. PCRs for the nc *H4/H*3 were performed with the proof‐reading Phusion High‐Fidelity DNA Polymerase (Thermo Fisher Scientific) in 25 µl volumes with 0.2 mM of each dNTP, 1 mM of each primer, 5 µl 5× Phusion HF Buffer, and Phusion DNA Polymerase (0.4 U). Phusion PCRs started with 30 s at 98°C, followed by 35 cycles with 10 s at 98°C, 10 s at 71°C, 30 s at 72°C, and a final extension for 7 min at 72°C. All PCRs were run on a Master gradient thermocycler (Eppendorf). Purification and direct sequencing (in both directions, using the PCR primers) were performed at LGC Genomics. If the complete H4/H3 sequence could not be obtained by direct sequencing, due to the large fragment size and/or the presence of different alleles in the variable IGS region, PCR products were cloned. In case that specimens were heterozygous and the IGS sections differed in more than a single insertion/deletion (indel), up to four clones were sequenced. Before cloning, PCR products were excised from 1% agarose gels and purified using the QIA quick Gel Extraction Kit (QIAGEN), extended by A‐endings with the DyNAzyme II DNA Polymerase (Thermo Fisher Scientific) and then cloned with the TOPO‐TA cloning kit (Thermo Fisher Scientific). Plasmid preparation and sequencing of the cloned fragments were performed at LGC Genomics using M13 universal primers.

### 2.5 Nucleotide sequence analyses

The raw forward and reverse sequences were manually aligned in BioEdit v.7.1.3 (Hall, 1999) and checked for errors. Prior to the phylogenetic analyses, sequences of the separate data sets were aligned, which was straightforward for the *COI* because there were no indels. The other sequences of *Schileykula* and *Sphyradium* were aligned with MAFFT v.7 (Katoh & Standley, 2013) using the option “E‐INS‐i” for alignments with multiple conserved domains and long gaps (Katoh, Kuma, Toh, & Miyata, 2005) and refined in BioEdit v.7.1.3 (Hall, 1999). Subsequently, all positions in the *12S*, *16S,* and H4/H3 alignments containing gaps were removed with trim‐al v1.2 (Capella‐Gutiérrez, Silla‐Martínez, & Gabaldón, 2009) using the “nogaps” option, followed by a second trimming step applying the “strictplus” algorithm (removal of highly saturated alignment regions). A second alignment including the H4/H3 sequences of *Schileykula* only was created for the calculation of median‐joining networks, applying the same trimming options for the IGS region as for the latter data sets.

Phylogenetic trees were reconstructed with three different sequence data sets containing (a) all 86 *COI* sequences of *Schileykula* and *Sphyradium*; (b) the concatenated *COI*, *12S*, and *16S* alignment of 41 individuals (*Schileykula*: 33, *Sphyradium*: 8); and (c) the H4/H3 alignment with 53 sequences (including additional clones of some specimens) of 43 individuals (*Schileykula*: 35, *Sphyradium*: 8). The latter two data sets differ in individual numbers, because in two specimens either the mt *COI* (IndID: 6256) or *12S* (7114) could not be amplified. Prior to the phylogenetic analyses, identical sequences in each of the three data sets were collapsed, resulting in 57 (a), 35 (b) and 42 (c) haplotypes. We did not calculate trees based on a concatenated data set combining mt and nc markers because the data sets partially give conflicting signals. We think this would only be justified if gene flow between lineages can be excluded.

Substitution models were selected separately for the alignments/partitions with JModelTest v.2.1.5 (Darriba, Taboada, Doallo, & Posada, 2012), based on the corrected Akaike information criterion (AICc). Information on sequence lengths, alignments, and the particular substitution models is provided in Table 3. Bayesian inference (BI) analyses were calculated with MrBayes v.3.2.1 (Huelsenbeck & Ronquist, 2001; Ronquist & Huelsenbeck, 2003), applying the substitution models best fit according to the AICc. Analyses for the three data sets were run for 10^7^ generations each (two runs each with four chains, one of which was heated), sampling every hundredth tree. The first 25% of trees were discarded as burn‐in and 50% majority rule consensus trees were calculated from the remaining 75,000 trees. Maximum likelihood (ML) analyses were performed with IQtree v.1.4.4 (Nguyen, Schmidt, Haeseler, & Minh, 2015) using the same substitution models and 1,000 bootstrap replicates each. In the BI and ML analyses, only the three sections of the combined mt data set were treated as separate partitions, whereas the H4/H3 alignment was not partitioned because of the low number of informative sites in the coding *H4* and *H3* regions. Mean Kimura 2‐parameter (K2P) distances between and maximum K2P distances within species/subspecies clades were calculated for the 86 sequence *COI* data set using Mega v.6.6 (Tamura, Stecher, Peterson, Filipski, & Kumar, 2013).

**Table 3:** Sequence data sets and alignments

Partition	Sequence length (bp)	Alignm. method (MAFFT)	Alignment length (bp)	Nogaps (trim‐al) (bp)	Strictplus (trim‐al) (bp)	Align. final (bp)	Subst. model (Akaike information criterion)
Alignments for phylogenetic tree inference							
*COI*	655	manual	655	—	—	655	HKY+I+G
*12S*	669–709	E‐INS‐i	763	563	500	500	GTR+I+G
*16S*	836–866	E‐INS‐i	915	747	628	628	GTR+I+G
*H4*/IGS/*H3*	271/435−537/346	E‐INS‐i	271/672/346	271/379/346	271/353/346	970	SYM+G
Alignment for *H4*/IGS/*H3* network calculations							
*H4*/IGS/*H3*	271/435−537/346	E‐INS‐i	271/647/346	271/389/346	271/364/346	—	—

In order to visualize potential recombination between nc H4/H3 variants, median‐joining networks were constructed for the three partitions (*H4*, IGS, and *H3*) with Network 5.0.0.1 (Fluxus Technology Ltd.) and post‐processed using the Steiner (MP) algorithm (Polzin & Daneshmand, 2003). Haplotype networks were graphically arranged with Network Publisher (Fluxus Technology Ltd.) and post‐edited in Adobe Photoshop CC v.2015.01 (Adobe Systems).

All sequences were uploaded to NCBI GenBank under the accession numbers MK332711–MK332746 (*12S*), MK332747–MK332782 (*16S*), MK332783–MK332862 (*COI*), and MK332863–MK332910 (H4/H3). Alignments are available as Alignments [Link], [Link], [Link], [Link].

## 3 RESULTS

### 3.1 Mitochondrial gene trees

**Figure 3:**
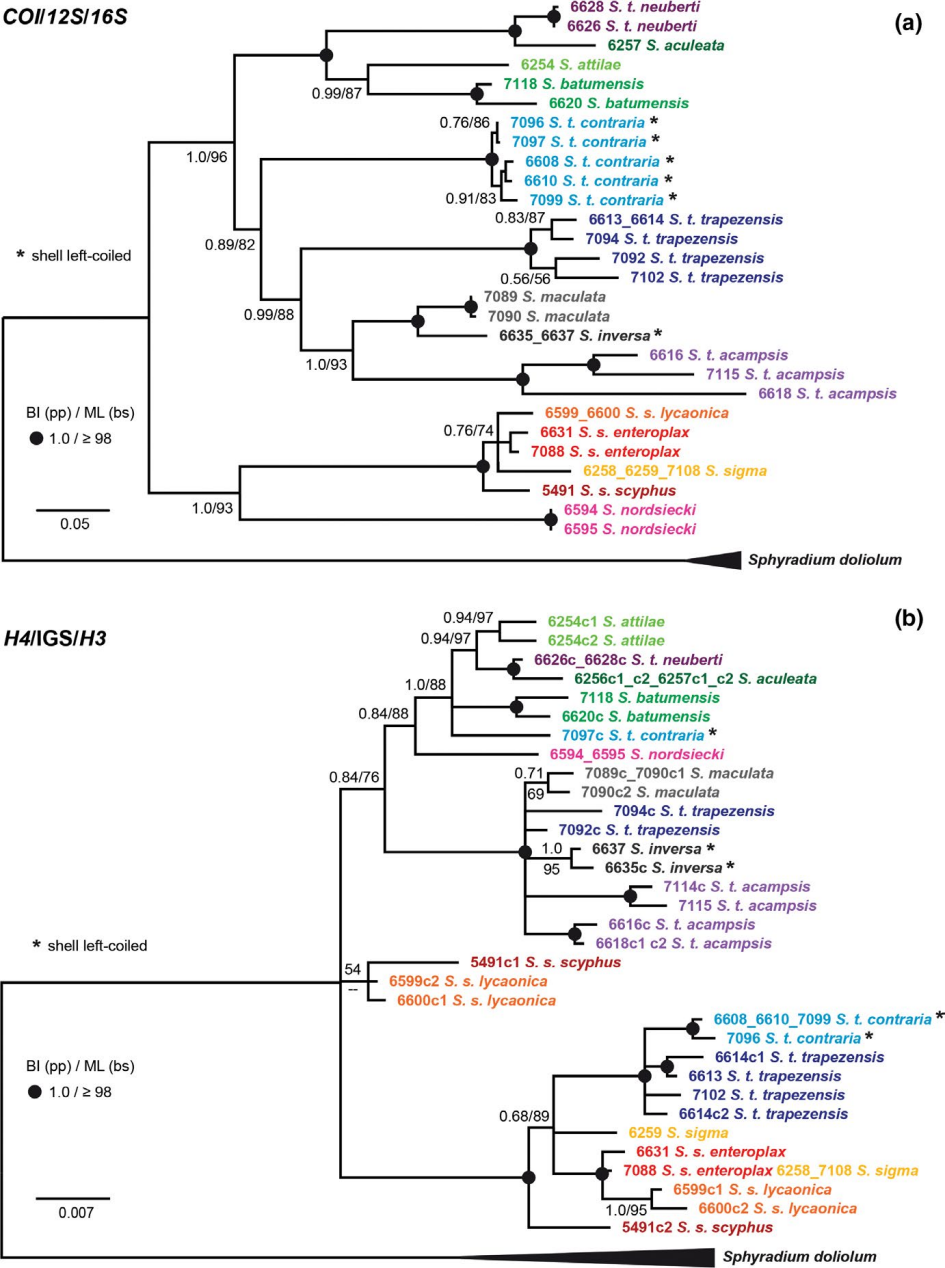
Phylogenetic trees reconstructed based on the alignments of the (a) combined mt *COI*, *12S*, *16S* nucleotide sequences and (b) the nc H4/H3 nucleotide sequences. The scale bars indicate the expected number of substitutions per site according to the models of sequence evolution applied. Black dots indicate nodes with high Bayesian inference posterior probabilities and ML bootstrap values (see figure). Specimens marked by an asterisk are sinistral. Cloned sequences in the nc tree are indicated by a “c” following the ID number

In the BI and ML trees based on the concatenated mt nucleotide sequences (*COI*, *12S*, *16S*), most relationships between the clades were well supported, although deeper nodes of the trees did not obtain maximum support (Figure 3a). In the following, we indicate BI posterior probability values (in decimals) and ML bootstrap support values (in %) in brackets when referring to the clades. The combined mt tree shows two main clades, one of which (1.0/93) bifurcates into the *S. nordsiecki* lineage and a subclade comprising *S. scyphus* and *S. sigma* (1.0/100). The latter subclade includes all three subspecies of *S. scyphus*, namely *S. s. scyphus*, *S. s. enteroplax,* and *S. s. lycaonica* as well as *S. sigma*. The second main clade (1.0/96) comprises the remaining *Schileykula* taxa included in the study. *Schileykula aculeata* is the sister lineage of *S. t. neuberti* (1.0/100) and their sister clade contains *S. attilae* and *S. batumensis* (1.0/87). The sister group relationship between *S. maculata* and *S. inversa* is highly supported (1.0/100). Together with *S. t. acampsis*, they form a clade with high support (1.0/93). *Schileykula t. trapezensis* forms the sister lineage of the latter clade with moderate support (0.99/88) and *S. t. contraria* branches off from a more basal node with low support (0.89/82). Thus, in the mt gene trees, the four subspecies of *S. trapezensis* represent distinct, polyphyletic lineages. Moreover, the two sinistral taxa *S. inversa* and *S. t. contraria* are located at distinct positions in the tree, strongly supporting independent evolution of right–left reversal.

In the BI and ML phylograms based on the *COI* nucleotide sequences only (including all samples of *Schileykula* and *Sphyradium*), deeper splits within *Schileykula* are not resolved (Figure S1). Similarly, as in the combined mt tree, the position of *S. sigma* within the clade containing the three subspecies of *S. scyphus* is not essentially supported (0.76/74). The K2P distances in the *COI* sequences between *Schileykula* taxa and the out‐group *Sp. doliolum* are provided in Table 4. The mean K2P distance between the *Schileykula* taxa and *Sp. doliolum* was 22.5%. The maximum intraspecific K2P distances were highest at 12.9% in *S. t. acampsis*, 7.1% in *S. t. trapezensis*, 6.5% in *S. batumensis*, and 2.5% in *S. s. enteroplax*. No genetic variability in the *COI* was found in *S. s. lycaonica*, *S. nordsiecki*, *S. inversa*, *S. attilae*, and *S. aculeata*. However, there is a sampling bias because of the latter taxa only a few specimens from single localities were investigated. In addition, we also provide the uncorrected *p*‐distances in Table S1.

**Table 4:** Mean Kimura 2‐parameter (K2P) distances in the *cytochrome c oxidase subunit 1* between taxa and maximum K2P distances within taxa

	1	2	3	4	5	6	7	8	9	10	11	12	13	14	15
Max dist.	9.7	0.2	—	1.1	2.5	—	12.9	0.5	—	7.1	2.0	6.5	—	—	0.5
*Sphyradium doliolum*															
*Schileykula scyphus scyphus*	20.9														
*Schileykula scyphus lycaonica*	22.8	5.5													
*Schileykula sigma*	22.3	7.5	7.4												
*Schileykula scyphus enteroplax*	23.3	5.1	5.4	6.4											
*Schileykula nordsiecki*	23.0	16.6	17.1	19.0	16.5										
*Schileykula trapezensis acampsis*	23.8	18.6	18.6	19.4	18.6	19.7									
*Schileykula maculata*	20.9	17.6	19.0	18.6	18.1	16.6	15.4								
*Schileykula inversa*	22.2	17.1	18.4	17.9	17.0	15.6	15.7	7.8							
*Schileykula trapezensis trapezensis*	20.9	17.3	18.4	17.6	18.2	16.2	16.1	12.2	13.8						
*Schileykula trapezensis contraria*	20.9	17.9	17.6	17.2	17.9	16.4	16.3	12.4	11.4	13.6					
*Schileykula batumensis*	23.6	18.8	19.2	18.1	18.8	16.4	18.3	12.9	14.0	15.2	14.0				
*Schileykula attilae*	23.7	16.8	17.8	17.8	17.3	16.0	17.2	12.4	13.7	14.6	12.7	10.7			
*Schileykula aculeata*	22.8	16.5	16.2	16.8	16.4	16.0	16.8	12.3	13.6	14.7	14.1	13.3	12.5		
*Schileykula trapezensis neuberti*	23.8	16.6	18.1	18.2	16.6	17.4	17.7	13.8	13.8	16.5	15.5	13.0	12.7	6.5	

### 3.2 Nuclear gene trees

Concerning the H3/H4 region, several individuals provided more than a single variant of the H4/H3 sequence section, which can be explained by the presence of distinct alleles at the two homologous chromosomes or the presence of distinct variants in the multi‐copy histone cluster. Of the 35 *Schileykula* individuals analyzed, 16 were homozygous in the H4/H3 region, ten each had two variants differing only in a single nucleotide position in the IGS, six showed two variants differing at two to three sites, and three had two variants differing at multiple sites.

The BI and ML trees of the *H4*/IGS/*H3* data set (Figure 3b) exhibit complex patterns, which substantially differ in topology from the combined mt gene tree (Figure 3a). Similarly, as in the mt tree, there are two main clades, however, with different taxon compositions. One main clade (1.0/100) contains sequences of all *S. scyphus* and *S. sigma* specimens analyzed and a subclade with sequences of most *S. t. trapezensis* (6613, 6614, 7102) and *S. t. contraria* (6608, 6610, 7096, 7099) specimens (1.0/100). Besides that, one specimen of *S. s. scyphus* (5491) and two specimens of *S. s. lycaonica* (6599, 6601) yielded in addition very different H4/H3 variants forming a short‐branched clade (54/—) at the base of the in‐group clade. All other sequences cluster in one weakly supported (0.84/76) clade. Within the latter, a well‐supported subclade (1.0/100) contains all sequences of *S. t. acampsis*, *S. inversa*, and *S. maculata* as well as the sequences of two specimens of *S. t. trapezensis* (7092, 7094). This subclade corresponds in taxon composition with one of the clades in the combined mt tree. *Schileykula t. neuberti*, *S. aculeata*, *S. attilae*, and *S. batumensis* are grouped in a subclade (1.0/88) together with one specimen of *S. t. contraria* (7097). *Schileykula nordsiecki* forms the sister lineage to the latter clade (0.84/88).

### 3.3 Median‐joining networks of *H4*, IGS, and *H3*

**Figure 4:**
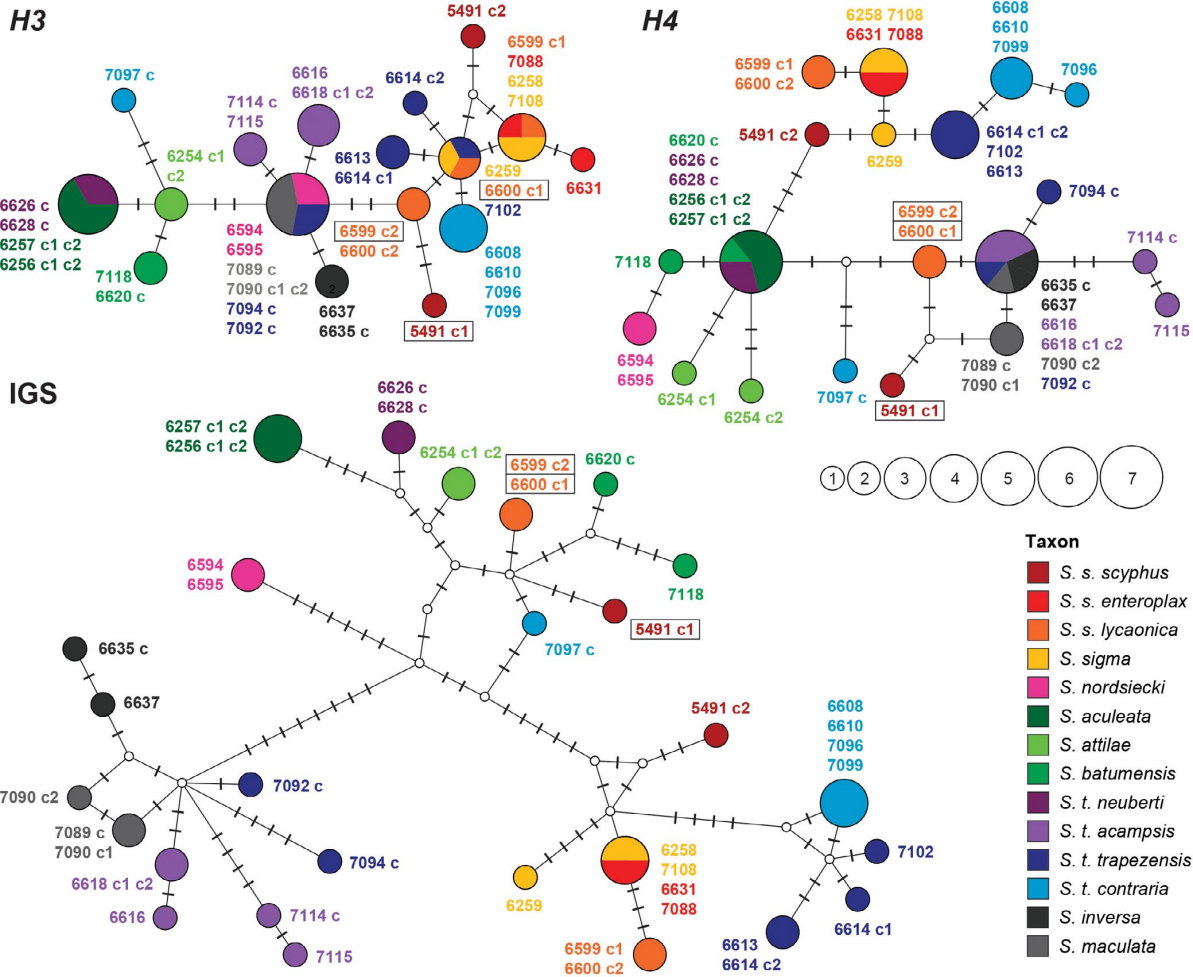
Median‐joining haplotype networks based on the alignments of the three sections of the nc H4/H3 sequences (*H4*, IGS, and *H3*) from *Schileykula*. The circle size corresponds to the number of sequences with identical haplotypes. Bars indicate the number of substitutions between nodes. The taxa are distinguished by color. Individual IDs are shown next to their haplotypes, with “c” symbols marking cloned sequences. Clones of the aberrant haplotypes from three *Schileykula scyphus* individuals are framed in black

While the BI and ML trees were generated with the complete H4/H3 region, median‐joining haplotype networks were calculated separately for the three sections (*H4*, IGS, *H3*) in order to visualize potential recombination products between different variants (Figure 4). In particular, the placement of three clones of *S. s. scyphus* (5491 c1) and *S. s. lycaonica* (6599 c2, 6600 c1) in the short‐branched clade at the base of the H4/H3 tree (Figure 3b) suggested that recombination might have happened. In the network, the *H3* sections of these cloned sequences cluster with the sequences from the other *S. scyphus* individuals. However, their IGS regions had similar sequences to those of *S. batumensis*, *S. attilae*, and *S. t. neuberti*, whereas the *H4* sections were similar to those of *S. t. acampsis*, *S. maculata*, *S. inversa*, and two *S. t. trapezensis* specimens (7092, 7094). The three haplotypes from *S. s. scyphus* (5491 c1) and *S. s. lycaonica* (6599 c2, 6600 c1) seem to be recombination products between the alleles in the two main clades. These three cloned sequences also differ in a unique 30 bp insertion at the 5′‐end of the IGS region (not visible in the networks). Moreover, in *S. nordsiecki* (6594, 6595), the placements of the three sections are not concordant. The *H3* sections are identical to the haplotypes from *S. maculata* (7089, 7090) and two *S. t. trapezensis* specimens (7090, 7092), whereas the *H4* sections are similar to those from *S. batumensis* (7118) and related species, and the IGS region is positioned between the two main haplotype clades.

## 4 DISCUSSION

We performed phylogenetic analysis on 18 species and subspecies of the land snail genus *Schileykula* using nc (H4/H3) and mt (*COI, 12S, 16S*) data sets, with several samples of the monotypic sister genus *Sphyradium* as out‐group. It is worth mentioning that the mt phylogeny roughly reflects anatomical characters: The taxa grouped in the two main clades in the mt tree each share similar anatomical traits with respect to the size and shape of the penial cecum (Figure S2). *Schileykula scyphus* and *S. nordsiecki* are characterized by a rather vestigial, slender penial cecum, whereas all other species possess a large cecum with widened basis (Hausdorf, 1996; Páll‐Gergely, 2011; Páll‐Gergely & Asami, 2013; Páll‐Gergely & DeMattia, 2016). On the other hand, the trees partly disagree with the morphology‐based classification asking for some taxonomic changes. Furthermore, the mt and nc trees show different patterns implying several hybridization events.

### 4.1 Hybridization

The nc phylogeny of *Schileykula* reveals complex patterns, which indicate at least two hybridization events between distinct taxa. The dextral *S. t. trapezensis* and the sinistral *S. t. contraria* are separated by a genetic K2P distance of 13.6% in the *COI* and do not form sister lineages in the combined mt tree (Figure 3a). In the nc H4/H3 tree (Figure 3b) most, but not all specimens of *S. t. contraria* and *S. t. trapezensis* form a highly supported subclade within the *S. scyphus* clade. The most parsimonious explanation for this pattern is that two specimens of *S. t. trapezensis* (7092, 7094) and one specimen of *S. t. contraria* (7097) carry original variants, whereas the H4/H3 variants in the other specimens originate from two hybridization events. In a first event, H4/H3 variants may have introgressed from *S. scyphus* into *S. t. trapezensis*. Their distribution ranges are adjacent in three regions (Figure 2). In a second hybridization event, the newly acquired H4/H3 variants were probably passed from *S. t. trapezensis* to the narrow ranged *S. t. contraria*. The mixed sequence pattern of the recombinant sequences as visible in the nc H4/H3 data set implies that hybridization and recombination might have occurred several times. The genuine and the recombinant H4/H3 alleles of three specimens (IndID 5491, 6599, 6600) were probably maintained in populations of *S. scyphus* over a long time because they are present in two geographically distinct subspecies (>400 km geographic distance) and differ in several nucleotide positions. The aberrant H4/H3 variants theoretically might represent a paralogous histone cluster, but this would be in conflict with its absence in other *Schileykula* taxa (if it was present, we would have expected to co‐amplify it, as it was the case with the three specimens). Moreover, the separate analyses of the three sections clearly indicate that these sequences are recombinants. Generally, multigene families such as those encoding histones and rRNA are subject to so‐called concerted evolution (Liao, 1999). The primary driving force for concerted evolution in tandemly repeated multigene families is probably intra‐chromosomal homogenization, whereas inter‐chromosomal genetic exchange is much rarer (Liao, 1999; Liao, Pavelitz, Kidd, Kidd, & Weiner, 1997; Schlötterer & Tautz, 1994). In two studies on the genus *Orcula* Held, 1837, the H4/H3 sequence section was analyzed in over a hundred specimens. Approximately 90% of the *Orcula* specimens were homozygous regarding the H4/H3 loci and only three provided more than two distinct variants (Harl, Duda, et al., 2014; Harl, Páll‐Gergely, et al., 2014), which were consequently interpreted as alleles of one histone cluster and not as paralogues.

Another possible explanation for some sequence patterns would be ancestral polymorphism in the H4/H3 genes. However, the nc haplotypes of *S. t. contraria* and *S. t. trapezensis*, which cluster with *S. scyphus*, are very similar to each other and also to *S. scyphus* (Figure 3b). Considering that these haplotypes were affected throughout time in the same way by mutations as those found in the other specimens, they should also be more distinct (have longer branches in the tree), which is not the case. The morphological similarity of *S. t. trapezensis* and *S. t. contraria* and the close vicinity of populations further support the assumption of introgression after secondary contact. However, genome‐wide nuclear sequence data would be required to confirm our assumption and to clarify the true extent of admixture between populations.

Geographically, hybridization between *S. trapezensis* and *S. scyphus* might have occurred particularly in the eastern Black Sea Region (Gümüşhane, Bayburt, Trabzon, and parts of Erzurum province), where their distribution ranges partly overlap. Hybridization might have occurred also in the north‐easternmost part of Turkey (Erzurum and Artvin provinces), where five *Schileykula* taxa are distributed in a relatively narrow range (see Figure 2). Neubert (1993) reported hybrid specimens in populations of *S. batumensis* and *S. t. acampsis*, which are the only *Schileykula* taxa known to occur in sympatry over a broad range. We only sampled a few specimens and localities each. Therefore, although the sequence data did not indicate mixing between these two taxa, analyzing a larger sample covering a wider area could reveal hybridization between them and/or other taxa.

#### 4.1.1 *Schileykula trapezensis* is polyphyletic

Following the current classification, *S. trapezensis* comprises four subspecies, all of which were included in the present study. They all inhabit north‐eastern Turkey, whereby *S. t. trapezensis* and *S. t. acampsis* each occupy relatively large areas spanning a distance of about 100 km. The other two subspecies have extremely narrow ranges and occur in parapatry with the latter two. The sinistral *S. t. contraria* was so far found only at three sites between populations of the dextral *S. t. trapezensis*, separated by a distance of 1 km only. *Schileykula t. neuberti* is known from a valley about 2 km east of the north‐eastern margin of the distribution area of *S. t. acampsis*. The mt and nc trees show that *S. trapezensis* is actually polyphyletic and the subspecies rather should be considered as distinct species. *Schileykula t. neuberti* is closest related to *S. aculeata* in both the mt and nc phylogenies (Figure 3), separated by a genetic K2P distance at 6.5% in the *COI* only; their sister group relationship is highly supported. *Schileykula t. acampsis* represents the sister group of *S. maculata* + *S. inversa* in the combined mt tree. Therefore, *S. t. acampsis* should be considered as a separate species independent from *S. t. trapezensis*. *Schileykula t. contraria* forms a quite distinct lineage in the combined mt tree. Despite some indications of past hybridization between *S. t. contraria* and *S. t. trapezensis* (the latter issue is discussed below), we suggest treating *S. t. contraria* as independent species.

#### 4.1.2 *Schileykula scyphus*, *S. nordsiecki*, and *S. sigma*

*Schileykula scyphus* shows the widest distribution of all *Schileykula* species. Only *S. nordsiecki* occurs within the western range of *S. scyphus*. It is only known from one single locality in south‐east Turkey, geographically located between populations of *S. s. lycaonica*. The shells of *S. nordsiecki* are indistinguishable from those of *S. s. lycaonica*, but the morphology of the genital anatomy is strikingly different, which resulted in its differentiation on species level (Hausdorf, 1996). Both the mt and nc phylogenies (Figure 3) provide clear evidence that *S. nordsiecki* is genetically distinct from *S. scyphus*. Although *S. scyphus* and *S. nordsiecki* are closest related to each other in the combined mt tree, they are separated by relatively high genetic K2P distances at 16.5%–19.0% in the *COI*, which is in accord with their proposed species status.

*Schileykula sigma* has been considered as an independent species because of its peculiar shell characters, in particular, the sigmoid formation of the columellar lamella (Hausdorf, 1996), the genital anatomy, however, is similar to that of *S. scyphus* (Páll‐Gergely, 2011). In the mt and nc phylogenies, *S. sigma* is nested within *S. scyphus*. Genetic K2P distances between the three subspecies of *S. scyphus* (5.1%–5.5%) are in a similar range as that between the latter and *S. sigma* (6.4%–7.5%). In order to clarify the status of *S. sigma*, that is, to test whether it is reproductively isolated from *S. scyphus* or not, hybridization experiments would be necessary. However, controlled laboratory experiments have not been performed with any *Schileykula* species so far. Given the similarities in the mt and nc genes analyzed as well as in the genital anatomy, we consider *S. scyphus* and *S. sigma* as conspecific.

### 4.2 Origin of sinistral taxa

The two sinistral species do not form sister groups in the nc and mt trees, suggesting that the change of coiling direction from dextral to sinistral happened two times independently within the genus. However, since our data indicate that hybridization affected several taxa (including *S. t. contraria*), it is possible that there was a single origin of sinistrality in this group, which has subsequently been obscured by hybridization events. Generally, Turkish Orculidae exhibit a comparably high proportion of sinistral taxa (Gittenberger et al., 2012). Sinistral snails are more frequent in high‐spired taxa, and opposite‐coiled specimens have less difficulties in mating than enantiomorphic pairs possessing flat or globular shells (Asami et al., 1998). Whether the changes in chirality might have contributed to the speciation of both *S. inversa* and *S. contraria* remains speculative.

### 4.3 Classical taxonomy versus molecular phylogeny

**Figure 5:**
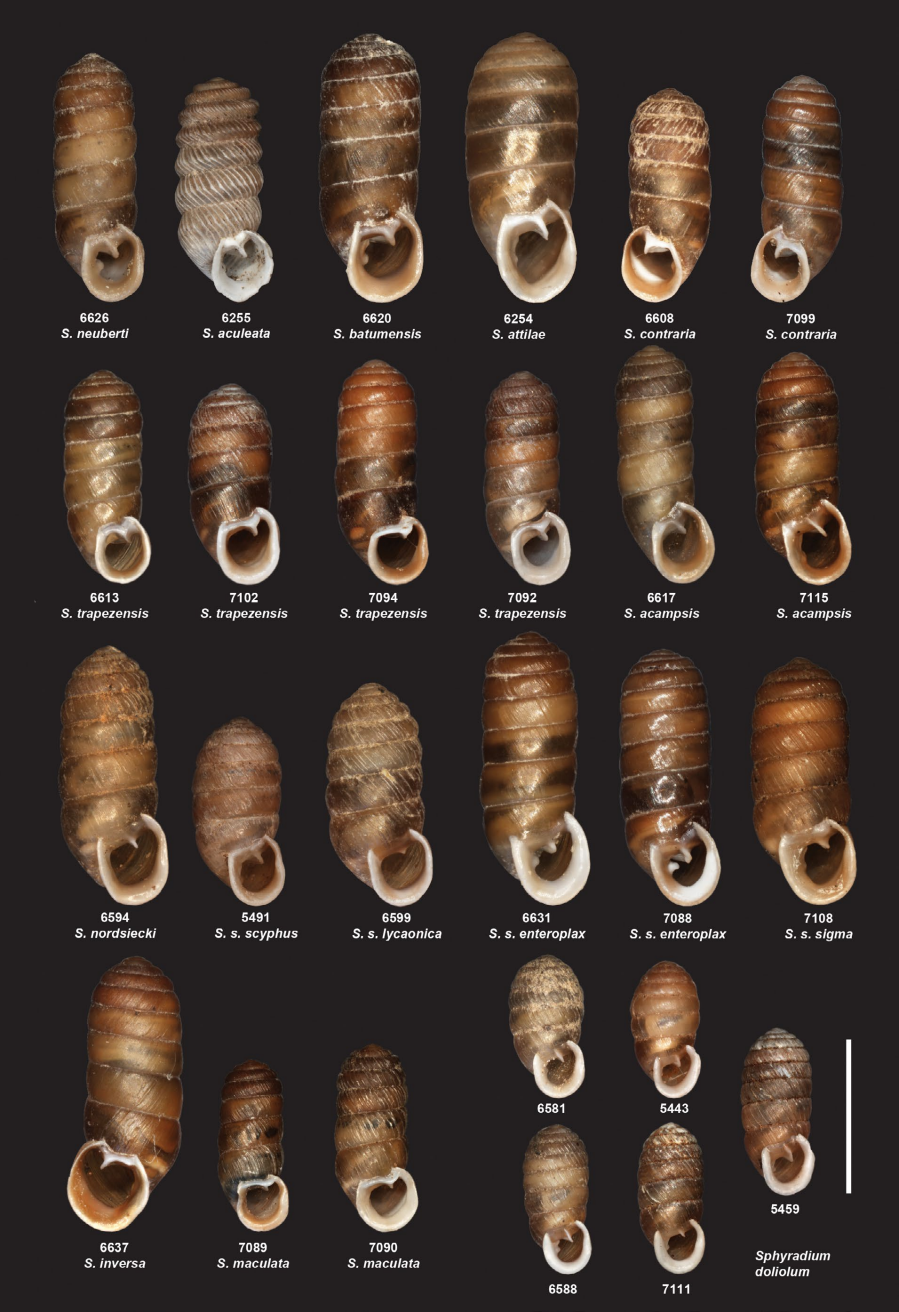
Adult shells of orculid taxa (*Schileykula* and *Sphyradium* spp.) of which nucleotide sequences were obtained in the present study. Numbers below the photographs are individual identification codes of each specimen that correspond with those in Table 1. The picture of *Schileykula aculeata* shows an empty shell from the same locality, because only juveniles were available for the molecular genetic analyses

The present study provides a phylogenetic basis for the systematics of *Schileykula* for the first time. Our DNA‐based analysis allowed us to re‐evaluate some pre‐existing hypotheses on the systematics within *Schileykula*. However, this revised systematics should be further tested using a higher sample size and optimally analyzing genome‐wide nuclear sequence data. Figure 5 shows selected specimens of the taxa studied.

*Schileykula aculeata* Gittenberger & Menkhorst, 1993

*Schileykula acampsis* Hausdorf, 1996

*Schileykula attilae* Páll‐Gergely, 2010

*Schileykula batumensis* (Retowski, 1889)

*Schileykula contraria* Neubert, 1993

*Schileykula inversa* Schütt, 1993

*Schileykula maculata* Páll‐Gergely & Asami, 2013

*Schileykula neuberti* Hausdorf, 1996

*Schileykula nordsiecki* Hausdorf, 1996

*Schileykula *(?) *robusta* (Nägele, 1906) (not included in study)

*Schileykula scyphus cilicica* Hausdorf, 1996 (not included in study)

*Schileykula scyphus crassa* (Pilsbry, 1922) (not included in study)

*Schileykula scyphus enteroplax* (Pilsbry, 1922)

*Schileykula scyphus erecta* Hausdorf, 1996 (not included in study)

*Schileykula scyphus lycaonica* Hausdorf, 1996

*Schileykula scyphus scyphus* (L. Pfeiffer, 1848)

*Schileykula scyphus sigma* Hausdorf, 1996

*Schileykula trapezensis* (Stojaspal, 1981).

## 5 CONCLUSION

The molecular genetic data presented here provide a first phylogenetic hypothesis based on a combination on mt and nc DNA sequences. Our results suggest some taxonomic revisions. Since *S. trapezensis* is polyphyletic, we propose treating the four subspecies as independent species. *Schileykula nordsiecki* was confirmed to represent an independent lineage distinct from *S. scyphus*. *Schileykula sigma* was shown to be closely related to *S. scyphus* and is therefore treated as a subspecies of the latter. The sinistral taxa *S. contraria* and *S. inversa* did not emerge as sister lineages in the mt and nc trees, implying that the change of the coiling direction might have happened two times independently. The incongruences between mt and nc trees suggest at least two independent hybridization events involving *S. contraria*, *S. scyphus*, and *S. trapezensis*. However, in order to shed more light on the complex patterns, future studies should include a larger number of samples from more localities as well as they would benefit from the analysis of additional nc markers.

## Supporting information


**FIGURE S1** Phylogenetic tree of the mt *COI* sequences obtained from *Schileykula* and *Sphyradium*.
**FIGURE S2** Reproductive anatomy of *Schileykula attilae* Páll‐Gergely, 2010 (left) and male part of the reproductive anatomy of *S. scyphus sigma* Hausdorf, 1996 (right) to highlight the differences of relative penial caecum sizes.
**TABLE S1** Uncorrected *p*‐distances of the *COI* sequences between taxa and maximum *p*‐distances between taxa.

**ALIGNMENT S1** Alignment of all *COI* sequences included in the study.

**ALIGNMENT S2** Alignment of *12S* rRNA sequences included in the concatenated mt tree.

**ALIGNMENT S3** Alignment of *16S* rRNA sequences included in the concatenated mt tree.

**ALIGNMENT S4** Alignment of H4/H3 sequences included in the nc tree.
