# Supplementary material for: Hybridization and recurrent evolution of left–right reversal in the land snail genus Schileykula (Orculidae, Pulmonata)
Source: J Zool Syst Evol Res. 2019 Dec 10;58(3):633–47. doi: 10.1111/jzs.12353 (PMC7540069; doi:10.1111/jzs.12353)
Supplement: Supplementary file 1 — FIGURE S1 Phylogenetic tree of the mt COI sequences obtained from Schileykula and Sphyradium. FIGURE S2 Reproductive anatomy of Schileykula attilae Páll‐Gergely, 2010 (left) and male part of the reproductive anatomy of S. scyphus sigma Hausdorf, 1996 (right) to highlight the differences of relative penial caecum sizes. TABLE S1 Uncorrected p‐distances of the COI sequences between taxa and maximum p‐distances between taxa. [file JZS-58-633-s001.pdf]

SUPPORTING INFORMATION

Hybridization and recurrent evolution of left-right reversal in the land snail genus *Schileykula* (Orculidae, Pulmonata)

Josef Harl, Elisabeth Haring, Barna Páll-Gergely

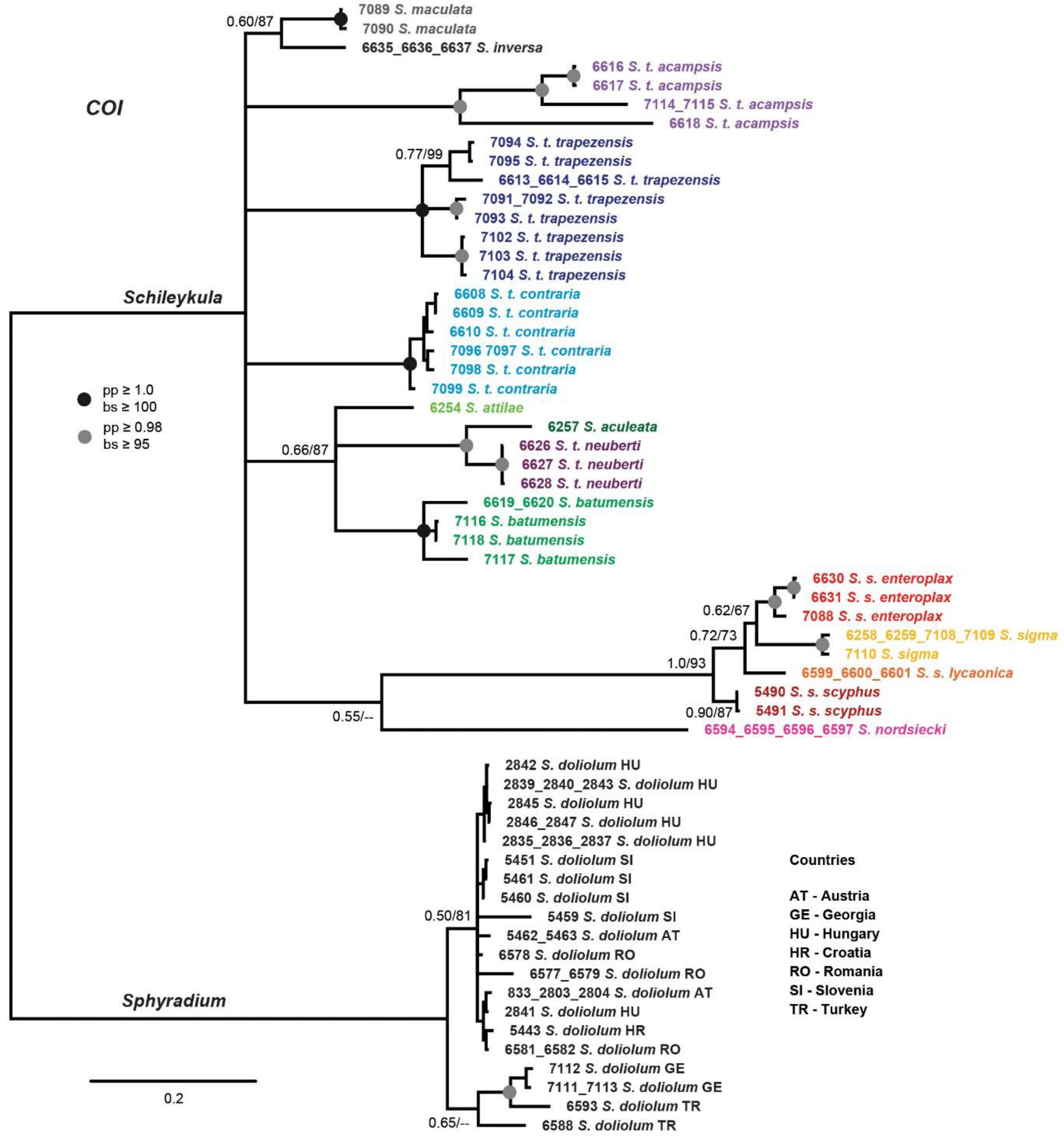

SUPPORTING FIGURE S1 Phylogenetic tree of the mt *COI* sequences obtained from *Schileykula* and *Sphyradium*. Black and grey dots indicate nodes with high BI posterior probabilities and ML bootstrap values (see figure). The scale bars indicate the expected number of substitutions per site according to the model of sequence evolution applied.

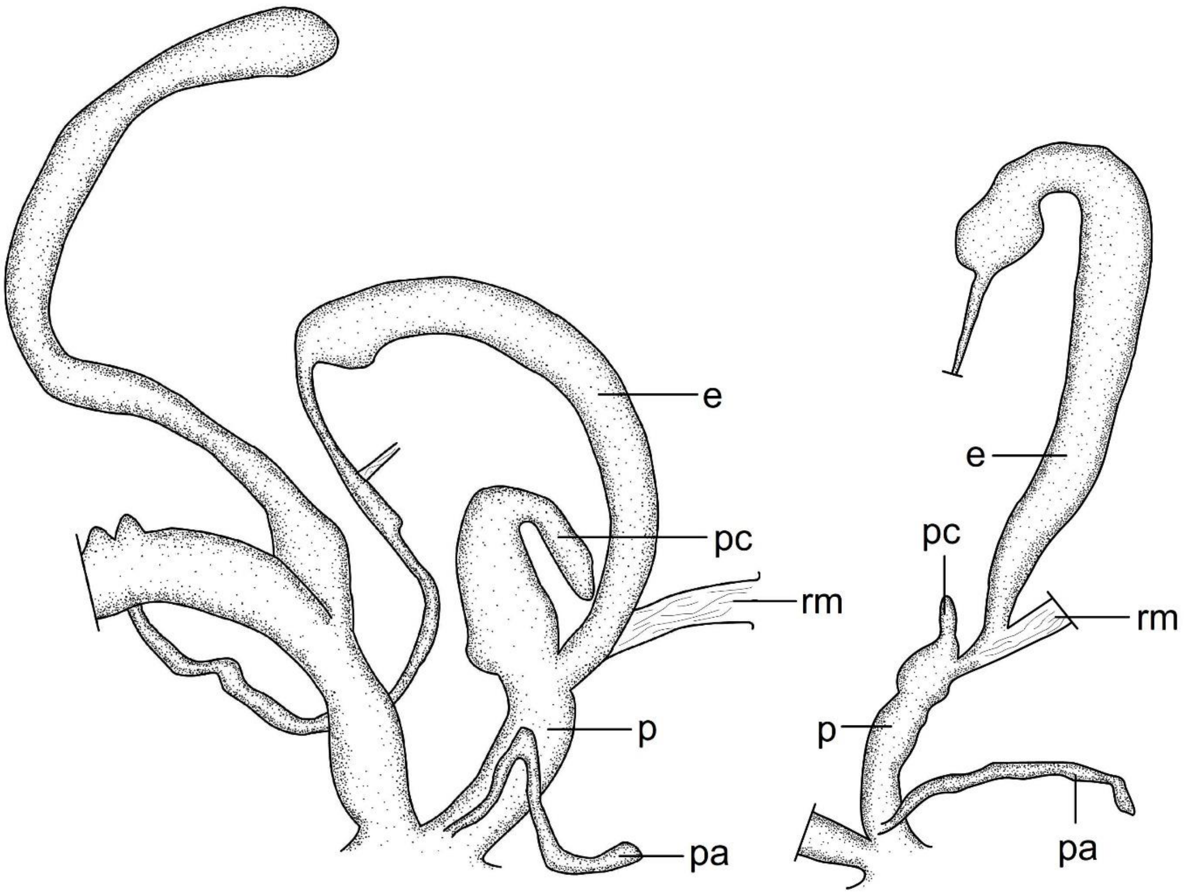

SUPPORTING FIGURE S2 Reproductive anatomy of *Schileykula attilae* Páll-Gergely, 2010 (left) and male part of the reproductive anatomy of *S. scyphus sigma* Hausdorf, 1996 (right) to highlight the differences of relative penial caecum sizes. Abbreviations: e: epiphallus, p: penis, pa: penial appendix, pc: penial caecum, rm: retractor muscle. Not to scale.

SUPPORTING INFORMATION TABLE S1 Uncorrected *p*-distances of the *COI* sequences between taxa and maximum *p*-distances between taxa.

	1	2	3	4	5	6	7	8	9	10	11	12	13	14	15
**max dist.**	9.7	0.2	-	1.1	2.5	-	12.9	0.5	-	7.1	2.0	6.5	-	-	0.5
Sp. doliolum															
S. s. scyphus	20.9														
S. s. lycaonica	22.8	5.5													
S. sigma	22.3	7.5	7.4												
S. s. enteroplax	23.3	5.1	5.4	6.4											
S. nordsiecki	23.0	16.6	17.1	19.0	16.5										
S. t. acampsis	23.8	18.6	18.6	19.4	18.6	19.7									
S. maculata	20.9	17.6	19.0	18.6	18.1	16.6	15.4								
S. inversa	22.2	17.1	18.4	17.9	17.0	15.6	15.7	7.8							
S. t. trapezensis	20.9	17.3	18.4	17.6	18.2	16.2	16.1	12.2	13.8						
S. t. contraria	20.9	17.9	17.6	17.2	17.9	16.4	16.3	12.4	11.4	13.6					
S. batumensis	23.6	18.8	19.2	18.1	18.8	16.4	18.3	12.9	14.0	15.2	14.0				
S. attilae	23.7	16.8	17.8	17.8	17.3	16.0	17.2	12.4	13.7	14.6	12.7	10.7			
S. aculeata	22.8	16.5	16.2	16.8	16.4	16.0	16.8	12.3	13.6	14.7	14.1	13.3	12.5		
S. t. neuberti	23.8	16.6	18.1	18.2	16.6	17.4	17.7	13.8	13.8	16.5	15.5	13.0	12.7	6.5	
